# Effects of a naturally occurring amino acid substitution in bovine PrP: a model for inherited prion disease in a natural host species

**DOI:** 10.1186/s13104-017-3085-8

**Published:** 2017-12-20

**Authors:** Catherine E. Vrentas, Justin J. Greenlee, Gregory H. Foster, James West, Marianna M. Jahnke, Mark T. Schmidt, Eric M. Nicholson

**Affiliations:** 1 United States Department of Agriculture, Agricultural Research Service, National Animal Disease Center, Ames, IA USA; 2 Department of Veterinary Diagnostic and Production Animal Medicine, College of Veterinary Medicine, Iowa State University, Ames, IA USA

**Keywords:** Prion, CJD, Creutzfeldt-Jakob disease, E200K, E211K, Cattle, *Bos taurus*, BSE, PrP, *PRNP*

## Abstract

**Objective:**

The most common hereditary prion disease is human Creutzfeldt-Jakob disease (CJD), associated with a mutation in the prion gene resulting in a glutamic acid to lysine substitution at position 200 (E200K) in the prion protein. Models of E200K CJD in transgenic mice have proven interesting but have limitations including inconsistencies in disease presentation, requirement for mixed species chimeric protein constructs, and the relatively short life span and time to disease onset in rodents. These factors limit research on the mechanism by which the mutation drives disease development. Therefore, our objective was to provide the first assessment of cattle carrying the homologous mutation, E211K, as a system for investigating longer-term disease mechanisms. The E211K substitution was associated with a case of bovine spongiform encephalopathy from 2006.

**Results:**

We assessed the molecular properties of bovine E211K prion protein, characterized the molecular genetics of a population of cattle E211K carriers (offspring of the original EK_211_ cow) in relation to findings in humans, and generated preliminary evidence that the impacts of copper-induced oxidative stress may be different in cattle as compared to observations in transgenic mouse models. The cattle E211K system provides the opportunity for future analysis of physiological changes over time.

**Electronic supplementary material:**

The online version of this article (10.1186/s13104-017-3085-8) contains supplementary material, which is available to authorized users.

## Introduction

### Background

Prion diseases are fatal diseases of the nervous system associated with misfolding of the prion protein (PrP^C^) into a more protease-resistant conformation (PrP^Sc^). In humans, a subset of disease is derived from the inheritance of mutations in the prion gene (*PRNP*). The most common form of hereditary Creutzfeldt-Jakob disease (CJD) is caused by a mutation in the prion gene (*PRNP*) resulting in a glutamic acid to lysine substitution at amino acid 200 (E200 K) [1–3]. However, the exact basis by which the E200K change leads to PrP^Sc^ accumulation and/or disease is unknown. The E200K change only slightly affects conformational stability of human recombinant PrP [4]. It has been proposed that an increase in hydrophobic surface exposure in E200 K protein contributes to aggregation and cellular toxicity [5].

Studies of human E200K carriers suggest a role for factors beyond simply the presence of the primary *PRNP* mutation in modulating CJD progression. In the Libyan Jewish population, there is a large variation in age of symptom onset among carriers (40–80+) [6]. Rates of CJD penetrance also vary between populations, from 67 to 96% [7, 8]. Notably, expression of the wild-type allele was higher in the majority of healthy middle-aged E200K carriers in a Libyan population, with an E:K transcript ratio of 50:1 in some individuals, whereas CJD-affected E200K heterozygotes primarily exhibited equivalent ratios of expression [9].

Recent work in a mouse model of E200K prion disease in which transgenic mice (carrying chimeric human/mouse *PRNP*) develop neurological disease and accumulate PrP^Sc^ suggests that oxidative stress synergistically interacts with the E200K mutation to induce disease. The E200K mutation impairs copper binding and is associated with increased copper sensitivity in cultured E200K fibroblasts and acceleration of disease in E200K mice dosed with copper [10]. Oxidative damage induced by reactive oxygen species generated by free copper ions [11] may also interact with the susceptibility of E200K PrP^C^ to be spontaneously oxidized at methionine residues [12]. Characteristics of other mouse models for inherited prion disease are reviewed in [13].

Until recently, no examples of naturally occurring, inherited prion disease were known to exist in non-human species. In 2006, a 10-year old crossbred (*Bos indicus* × *Bos taurus*) United States beef cow was diagnosed with H-type BSE. This animal possessed a non-synonymous polymorphism in *PRNP*, resulting in the change of amino acid 211 from glutamic acid to lysine (E211K) [14]. As E211 is homologous to the human E200 residue, this animal was proposed to represent the first example of a genetically-based TSE in livestock. A female offspring of this BSE case also carried the E211K mutation [15].

### Rationale

In order to develop therapeutic strategies against inherited prion diseases, an understanding of specific mechanisms resulting in disease development over time is important. Due to the long-term nature of many environmental stressors such as accumulation of oxidative damage in tissues, an experimental system is needed that allows for pre-symptomatic investigation in a species that is a natural host of prion diseases and has a lifespan significantly longer than that of a laboratory mouse. Therefore, here, we provide an initial characterization of the first natural host experimental system for genetic prion disease: heterozygous carriers of the analogous mutation in cattle PrP, E211K. Specifically, we consider characteristics of EK_211_ cattle that are not currently exhibiting disease signs (2–4 years of age), as a means of understanding preclinical phenotypes that may contribute to disease pathology in mammals with the 200K/211K mutation.

## Main text

### Origin and propagation of E211K cattle herd

The EK_211_ female offspring, a *Bos indicus* crossbred animal described in detail in [15], was used to produce a small herd of heterozygous cattle by superovulation and embryo transfer, which remains under observation at the USDA. EK_211_ animals were bred by artificial insemination to generate the first KK_211_ (homozygous mutant) calf in 2013. Sibling wild-type calves (EE_211_ homozygotes) were produced and characterized in parallel.

In the E200K mouse model, mice carrying the mutant allele on either a wild-type or null *PRNP* background exhibited onset of terminal illness at approximately 7 months of age [16], about a quarter of the life span of mice with the C57BL/6 J background (Jackson Laboratories). The disease endpoint of the 2006 BSE case represents approximately half the maximum lifespan of beef cattle (≈ 20 years), consistent with humans who develop disease in middle/late middle age. None of the monitored EK_211_ cattle have yet exhibited neurological signs.

### Evaluation of E211K protein folding and stability

**Fig. 1:**
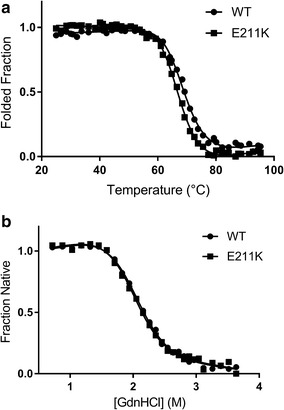
Comparison of wild-type and E211K bovine prion protein properties. **a** Thermal denaturation of wild-type and E211K bovine recombinant prion proteins. T_m_ results were consistent across multiple independent recombinant PrP preparations. Data points represent the results of a representative experiment (mean thermodynamic parameters for each mutant ± standard deviation, across 4–7 replicate curves, are provided in the text). 95% confidence intervals for the T_m_ were 68.3–68.7 °C (wild-type) and 65.6–65.9 °C (E211K). **b** GdnHCl denaturation of wild-type and E211K bovine recombinant prion proteins at 23 °C. A reduction in signal was noted between 0 and 0.6 M GdnHCl, which we propose is due to disruption of aggregative interactions, due to the long unstructured N-terminus on this version of the bovine PrP^C^ protein in particular; however, an initial baseline was well-defined between 0.6 and 1.4 M GdnHCl, which was used for the curve fitting. Mean thermodynamic parameters ± standard deviation are provided in the text for each protein

In order to evaluate the properties of the bovine E211K protein as compared to the human E200K protein, we compared stability characteristics of wild-type (glutamic acid residue at position 211) bovine recombinant PrP^C^ to E211K bovine recombinant PrP^C^ (representing residues 25–241; see Additional file 1 for detailed methods). When loss of secondary structure upon melting was monitored by loss of circular dichroism signal at 222 nm, the T_m_ (temperature at the unfolding midpoint) of bovine E211K protein (65.7 ± 0.2 °C) was lower than that of the wild-type protein (68.5 ± 0.3 °C; Fig. 1a), similar to previous findings for N-terminally truncated versions of recombinant human E200K (T_m_ = 67.0 ± 0.9 °C) and wild-type (T_m_ = 70.2 ± 0.1 °C) proteins [4]. Differences in ∆H_m_ (change in enthalpy upon unfolding determined at the midpoint of unfolding) between wild-type and E211K bovine PrP were unremarkable (∆H_m_ (wild-type) = 63.6 ± 5 kcal mol^−1^; ∆H_m_ (E211K) = 66.3 ± 2 kcal mol^−1^).

Using guanidine hydrochloride (GdnHCl) unfolding at 23 °C, the ΔG_H2O_ value of unfolding (measurement of the thermodynamic stability of the protein) for wild-type bovine protein was 5.0 ± 0.2 kcal mol^−1^ (m value = 2.3 ± 0.1 kcal mol^−1^M^−1^; [D]_1/2_ = 2.2 ± 0.1 M GdnHCl), within error of that determined for E211K bovine protein (∆G_H2O_ = 4.9 ± 0.1 kcal mol^−1^, m value = 2.4 ± 0.04 kcal mol^−1^M^−1^; [D]_1/2_ = 2.1 ± 0.01 M GdnHCl) (Fig. 1b). Similarly, for human full-length recombinant protein, ΔG_H2O_ values for wild-type and E200K proteins were not significantly different at 22 °C [17]. At 39 °C (cattle body temperature), we did observe higher stability of the wild-type (∆G_H2O_ = 5.0 ± 0.4 kcal mol^−1^; m-value = 2.9 ± 0.2 kcal mol^−1^M^−1^; [D]_1/2_ = 1.7 ± 0.1 M GdnHCl) as compared to E211K (∆G_H2O_ = 3.9 ± 0.1 kcal mol^−1^; m-value = 2.6 ± 0.1 kcal mol^−1^M^−1^, [D]_1/2_ = 1.6 ± 0.03 M GdnHCl) bovine prion protein. Overall, the relative properties of the bovine wild-type and mutant proteins are consistent with those of the human pair of proteins [4].

### Molecular genetics of EK_211_ cattle

Previous findings suggested a role for gene regulation in modulating development of prion disease in E200K CJD [18]. Such a modulation in cattle could be regulated through a 23 base pair (bp) insertion/deletion (indel) polymorphism in the promoter region and a 12 bp indel in exon 1 of *PRNP*. These sites have been implicated in BSE, where deletion at each site was over-represented in BSE cases as opposed to healthy animals, presumably by modulation of *PRNP* expression [19, 20]. We characterized the promoter region in EK_211_ animals via analysis of genomic DNA from the offspring of the U.S. 2006 case, which was heterozygous for both the 23 bp and the 12 bp indels. To identify the haplotype associated with K_211_, a single ovum was isolated from the offspring, and its genome was amplified. Sequencing of *PRNP* revealed the ovum was E_211_, and sizes of *PRNP* PCR products indicated that DNA from the ovum carried insertions at both the 23 and 12 bp indel sites. Therefore, the K_211_ allele must be associated with the 23 and 12 bp deletions. Upon birth of the first KK_211_ calf, its *PRNP* promoter was sequenced, confirming the haplotype of the K_211_ allele to be deletions at both sites.

Next, we examined whether any of the heterozygous EK_211_ cattle exhibited disparate levels of expression of the E and K alleles, as was observed in white blood cells of many middle-aged healthy human carriers [18]. One hypothesis is that an imbalance in allelic expression (E > K) earlier in the lifespan delays disease progression. Peripheral blood leukocytes (PBLs) were used as a non-invasive source of mRNA; this cell population has been demonstrated to express appreciable PrP^C^ in cattle [21]. The E:K allelic ratio was determined by sequencing of PCR-amplified products from reverse-transcribed RNA pools from three EK_211_cattle 4 years in age, utilizing allele quantification software (Additional file 1). The striking overexpression effect of the wild-type (E) allele in healthy human E200K carriers was not observed for any of these cattle samples (Table 1).

**Table 1:** Summary of characterization of properties of EK_211_ cattle

1	2	3	4	5	6	7	8
Calf number	Genotype	% WT Allele (G)/% K Allele (A)	C_t_ (*PRNP*)	C_t_ (*ACTB*)	ΔC_t_	Plasma TBARS: OD_540_ reading	Plasma SOD level (U/ml)
#79	EE_211_	–	30.4	19.6	10.8	0.18	0.036
#84	EK_211_	54/46	30.0	19.0	11.0	0.19	0.040
#85	EE_211_	–	30.0	19.2	10.8	0.15	0.046
#86	EE_211_	–	30.9	20.3	10.6	0.20	0.046
#87	EK_211_	54/46	30.0	18.9	11.2	0.20	0.055
#88	EK_211_	52/48	30.6	18.9	11.6	0.17	0.054
EE_211_ Average					*10.8* *±* *0.1*	*0.18* *±* *0.03*	*0.043* *±* *0.007*
EK_211_ Average					*11.3* *±* *0.3*	*0.19* *±* *0.02*	*0.052* *±* *0.008*

**Column 3** presents the results of allele expression analysis of RNA from EK_211_ cattle PBLs. Ratios were assessed by analysis of sequencing reactions of PCR amplicons of the region around codon 211 in *PRNP* cDNA, using QSVAnalyzer as described in additional methods (Additional file 1). Cattle were 48 months of age at the time of this analysis. **Columns 4–6** represent the results of qRT-PCR analysis of *PRNP* expression levels in both EE_211_ and EK_211_ cattle PBLs, with **Column 6** indicating the ΔC_t_ = C_t_(*PRNP*) − C_t_(*ACTB*). **Column 7** depicts results of TBARS readings on peripheral blood plasma samples. TBARS numbers reflect the OD_540_ of the cattle plasma samples as measured by the TBARS Kit from Cayman Chemical; due to interference from a separate absorbance peak in these samples, values are reported here as absorbance at 540 nm as opposed to a quantitative value based on the standard curve. **Column 8** depicts the results of SOD (Superoxide Dismutase) assays on peripheral blood plasma samples. SOD values are expressed in U/ml as derived from a standard curve generated with the SOD Assay Kit from Cayman Chemical. For columns 4–8, animals were 26 months of age at the time of analysis, with the exception of animal #79, which was 32 months at the time. The bottom two rows depict average values across biological replicates for parameters, expressed ± the 95% CI (confidence interval) in each case. (We note that we assume a normal distribution to calculate the CI; due to the unique nature of this cattle population, a small number of EK_211_ animals are available for testing, precluding more in-depth examinations of normality). The SOD assay on peripheral blood plasma was also completed on the offspring of the U.S. 2006 H-type BSE case (level = 0.057), and this value was included in the EK_211_ cattle average (**Column 8**)

Healthy human E200K carriers also exhibit higher overall *PRNP* expression as compared to both EE_200_ individuals and affected EK_200_ individuals, leading to the suggestion that increased wild-type allele expression could have a protective effect [18]. To determine if these *PRNP* expression characteristics were also observed in EK_211_ heterozygous cattle, we measured the level of overall *PRNP* gene expression in each of three EK_211_ cattle (2–3 years of age) as compared to the level in each of three age-matched EE_211_ cattle. PBLs were again utilized as a non-invasive source of cells. The level of *PRNP* expression (relative to *ACTB*; ΔC_t_) was only 5% higher in EE_211_ as compared to EK_211_ animals (Table 1; Additional file 2), consistent with very small differences in expression between the groups.

### Assessing oxidative stress in EK_211_ cattle

**Fig. 2:**
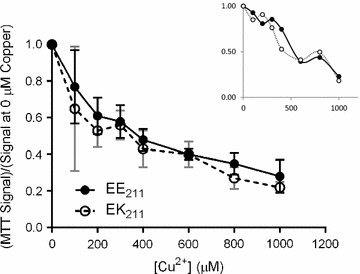
Test of copper sensitivity in PBLs from wild-type and EK_211_ cattle. PBLs isolated from each calf were treated with increasing concentrations of Cu^2+^ for 48 h, and cell viability was determined by the MTT assay. MTT values from triplicate wells for each animal were averaged, followed by averaging across genotypes to generate the displayed curves (the animals used were the same 6 as in Table 1, with the same bleed dates); data are displayed as the average MTT reading as a fraction of the average reading at 0 µM Cu^2+^, and the error bars represent the 95% CI for the calves of each genotype. Inset: Comparison of single KK_211_ calf (filled circles) to EE_211_ calf (open circles). Data analysis and axis labels are identical to the main panel

Finally, we utilized our cattle herd to examine the proposal that mammals carrying the E200K/E211K mutation are subject to increased oxidative stress. Mutant mouse fibroblasts have an increased sensitivity to copper in cell culture [10]. Our qRT-PCR results demonstrate expression of *PRNP* RNA in PBLs, confirming previous work demonstrating that *PRNP* expression in cattle PBLs is as high as that in fibroblasts [21]. PBLs isolated from EK_211_ and EE_211_ cattle were treated with copper concentrations (selected to correspond to the range in [10]), followed by measurement of cell viability by MTT assay. We did not observe a difference in copper toxicity, and by extension the level of metal-induced stress experienced, between genotypes (Fig. 2). The profile of copper sensitivity of cells from a KK_211_ calf was comparable to that for an EE_211_ calf tested in parallel (Fig. 2, inset), suggesting that the increased mutant copper sensitivity observed in humans is not recapitulated in this cattle system.

Finally, to determine if the E211K polymorphism is causing a detectable level of general oxidative stress in preclinical cattle, we analyzed peripheral blood samples for molecular markers of systemic oxidative stress. Levels of superoxide dismutase (SOD) in blood plasma were comparable between EE_211_ and EK_211_ cattle that were 2–3 years of age (Table 1); a 20% increase was observed in the EK_211_ group, but this difference did not reach the level of statistical significance (Additional file 2). A colorimetric TBARS assay, which detects lipid peroxidation resulting from oxidative stress, was also performed on the plasma samples. No genotype-dependent differences were noted (Table 1). Therefore, while it is possible that signs of oxidative damage in the blood will increase over time, young, preclinical cattle heterozygotes do not exhibit large increases in blood markers of systemic oxidative stress (Additional file 2).

## Limitations

This study describes the first natural host experimental system for genetic prion diseases, including molecular characterization of bovine E211K recombinant prion protein and molecular genetic experiments on EK_211_ cattle.

Limitations include the small sample size of these (rare) cattle, therefore limiting the ability to assess normality of parameters and limiting statistical power. However, in Additional file 2, we present t test comparisons and predictions of statistical power to provide information about the magnitude of differences in physiological parameters predicted to be detectable in further experiments with this herd. Additionally, unlike the case of the E200K prion mouse model (for which many *PRNP*-null mice exist), *PRNP*-null bovine cells were not available for comparative analysis.

Future characterization of the cattle over time, including analysis of parameters assessed in this study coupled with analysis of necropsied animals, will afford us the ability to assess molecular mechanisms of pathology. For example, brain tissue samples may reveal evidence of oxidative damage not perceivable in peripheral blood samples.

## Additional files

**Additional file 1.** Additional methods. More detailed information about methodologies used to generate data in Fig. 1, Fig. 2, and Table 1, including primers, qRT-PCR amplification conditions, and cycling information, as well as information about cell assays and recombinant prion protein purification.

**Additional file 2.** Additional Statistical Analysis. Additional description of statistical tests completed on the data, potential statistical limitations and their implications, and prospective statistical power analyses for assessment of effects of the 200K mutation.

## Abbreviations

*PRNP*: prion gene
CJD: Creutzfeldt-Jakob disease
PrP: prion protei
T_m_: temperature at the unfolding midpoint
ΔH_m_: change in enthalpy upon unfolding determined at the midpoint of unfolding
bp: base pair
PBLs: peripheral blood leukocytes
SOD: superoxide dismutase
