# Supplementary material for: Effects of a naturally occurring amino acid substitution in bovine PrP: a model for inherited prion disease in a natural host species
Source: BMC Res Notes. 2017 Dec 20;10:759. doi: 10.1186/s13104-017-3085-8 (PMC5738711; doi:10.1186/s13104-017-3085-8)
Supplement: Supplementary file 1 — Additional file 1. Additional methods. More detailed information about methodologies used to generate data in Fig. 1, Fig. 2, and Table 1, including primers, qRT-PCR amplification conditions, and cycling information, as well as information about cell assays and recombinant prion protein purification. [file 13104_2017_3085_MOESM1_ESM.docx]

**Additional File 1: Supplemental Methods**

***Expression and purification of recombinant prion protein***

Wild-type and E211K PrP expression vectors (untagged mature form of bovine prion protein, amino acids 25-241) were generated by PCR amplification of cDNA from cattle of each genotype and insertion of the product into a pET28 vector, similar to as previously described [1]. Briefly, recombinant (untagged) PrP proteins were overexpressed in *E. coli* BL21(λDE3) cells by growth of bacteria to an OD_600_ of 0.5-0.8, induction of expression with 1 mM IPTG, and harvest of cells after 16 hr. of growth at 37 ⁰C. Unless otherwise indicated, chemicals used in the purification were obtained from Sigma.

Purification of PrP proteins (both wild-type and E211K) was performed as described in [1]. Briefly, PrP inclusion bodies were recovered by lysis of bacterial cell pellets by sonication and centrifugation, and then inclusion bodies were solubilized in a 6 M guanidine hydrochloride buffer for 1 hr. and applied to nickel affinity resin (Qiagen). Proteins retained on the column were washed and eluted in buffer containing 8 M urea (Method 2 in [1]; eluted with addition of 300 mM imidazole + 10 mM β-mercaptoethanol). PrP proteins were refolded by slow (overnight) dialysis, first by dialysis into buffer with 8 M urea and no β-mercaptoethanol, and next into potassium phosphate buffer (no urea). Protein concentrations were measured by the Bio-Rad Protein Assay (BioRad #500-0006), and protein purity was assessed by SDS-PAGE.

***Analysis of protein conformation and stability***

Protein preparations were centrifuged at maximum speed in a microcentrifuge for > 15 minutes before circular dichroism (CD) analysis to remove particulates, as the full-length protein has a tendency to aggregate over time at 4 °C. All CD measurements were in 10 mM potassium phosphate (pH 7.0) buffer in a Jasco J-815 CD spectrometer with a Peltier temperature control. For thermal unfolding (Fig. 1A), PrP samples in 10 mM potassium phosphate buffer (pH 7) were heated in a 0.5 mm quartz cuvette, and the CD signal was monitored at 222 nm to detect loss of secondary structure. The rate of heating was 1° C/minute from 25 °C to 95 °C, with sampling each 1.5 °C and equilibration across ± 0.5 °C of each temperature. To calculate T_m_ (temperature at the unfolding midpoint), curves were fit with the equation in [2] in SigmaPlot (Systat Software, San Jose, CA) (Equation II, two-state monomer transition with correction for pre- and post-linear changes in ellipticity as a function of temperature). Thermal denaturations were reversible, with 85-90% of the original signal at 222 nm present after cooling. The ΔH_m_ (change in enthalpy upon unfolding determined at the midpoint of unfolding) values were calculated for each thermal unfolding curve using equation II in [2].

For guanidine denaturation experiments, PrP samples in 10 mM potassium phosphate buffer (pH 7.0) were placed in a 0.2 mm quartz cuvette at the indicated temperature, and the concentration of guanidine hydrochloride (GdnHCl) in the cuvette was increased by titration with concentrated GdnHCl solution. The GdnHCl stock used in chemical denaturation experiments was prepared in 10 mM potassium phosphate, and the concentration was calculated with the use of refractive index as described in [3]. Data collection followed mixing and equilibration. The CD signal was then corrected for dilution of the protein concentration. In our hands, equilibration was observed to occur rapidly, as was expected based upon published reports [4]. Thermodynamic parameters describing the chemical denaturation were determined using the equation II in [5] for curve fitting in SigmaPlot.

***Molecular genetics experiments***

For promoter haplotyping, the whole genome of a single ovum recovered from during the superovulation phase of EK_211_ herd production was amplified using the REPLI-g Whole Genome Amplification Midi kit (Qiagen), followed by PCR amplification of 130-202 base pair (bp) regions of the genome surrounding the 23 bp and 12 bp promoter indels and separation on 4% MetaPhor agarose gels as described previously [6]. Sequencing of the 211 codon was performed on a PCR-amplified product using primers F1 (5'-GCTGATGCCACTGCTATG-3') and F2 (5'-CACGGTCAATGGAACAAAC-3').

For *PRNP* analysis, peripheral blood leukocytes (PBLs) were isolated from blood samples collected from the jugular vein of cattle in acid-citrate-dextrose tubes. Briefly, the buffy coat was recovered by centrifugation of blood samples at 2500 X g for 20 minutes. Buffy coats were lysed in 20 ml ice-cold lysis buffer (10.57 mM Na_2_HPO_4_, 2.68 mM NaH_2_PO_4_, pH 7.2) for one minute, followed by the addition of 10 ml ice-cold restoring solution (11 mM Na_2_HPO_4_, 3 mM NaH_2_PO_4_, 0.5 M NaCl, pH 7.2). Samples were centrifuged at 1500 X g for 20 minutes, and lysis and centrifugation steps were repeated. PBLs were resuspended in PBS and counted using a hemocytometer. RNA was isolated from PBLs with the RNeasy Mini Kit (Qiagen) with an on-column DNase treatment step, and RNA quality was confirmed in all samples by analysis of rRNA by agarose gel electrophoresis. Reverse transcription was performed with the Superscript III RT kit with oligo (dT) priming (Life Technologies).

To determine allelic ratios, *PRNP* was PCR amplified from reverse-transcribed DNA derived from EK_211_ PBL RNA using forward primer X2/X3 (located at the Exon 2-Exon 3 boundary; 5’-GAATCACAGCAGATATAAGTCATC-3’) and reverse primer R1 (5’-AATGAGACACCACCACTACG-3’), and sequenced using primer F2 (above). The sequencing reactions were analyzed for the fraction of wild-type (GAA) and mutant (AAA) alleles at codon 211 of the nucleotide sequence using QSVanalyzer [7], with EE_211_ and KK_211_ sequences obtained with primer F2 as references.

To quantify *PRNP* expression levels, qPCR was performed using reverse-transcribed RNA from PBL samples and *PRNP* (AJ298878) and *ACTB* (NM_173979) primers previously validated for use on cattle samples [8]. Efficiency of *PRNP* and *ACTB* reactions were also independently confirmed via a dilution series (105% in each case). The C_t_ values for *PRNP* and *ACTB* gene expression, and the associated ΔC_t_ value for each calf, were determined by SYBR Green PCR (Life Technologies) on a 7500 Applied Biosystems Real-time cycler. Cycling was performed with SYBR Select Master Mix (with AmpliTaq DNA polymerase, SYBR GreenER^TM^, ROX^TM^, and UDG) following kit instructions (Life Technologies). Cycling conditions were as described in [8] and data was analyzed with the associated 7500 software. PCR product size and specificity, including the absence of product amplified from potential contaminating DNA, were confirmed by agarose gel electrophoresis of amplified reactions. qPCR reactions were performed with 1:100 dilutions of reverse-transcribed DNA, and C_t_ values for each animal reflect the average of 3 technical replicates of qPCR reactions. Dissociation curves were performed on each completed qPCR reaction and consisted of single peaks in each case.

***Copper sensitivity assays***

PBLs isolated from each calf (described above) were suspended in RPMI medium lacking phenol red (Life Technologies) + 10% Fetal Bovine Serum + 1% penicillin-streptomycin (Gibco/ThermoFisher) in a 96-well tissue-culture plate (Corning), with increasing concentrations of Cu^2+^ (from 0-1000 µM) added in triplicate in the form of CuSO_4_. Copper concentrations were chosen to match those used in the comparable experiments done in the mouse model [9], as opposed to the creation of a standard dose-response curve. Final cell seeding concentrations were 1-5 X 10^6^ per well. Plates were incubated at 37 ⁰C and 5% CO_2_ for 48 hours, at which point cell viability was measured with the DMSO quick version of the Vybrant MTT assay (Life Technologies). Both adherent and suspended cell types were retained, and absorbance was measured on a SpectraMax plate reader with subtraction of an MTT-only blank.

***Oxidative stress assays***

Superoxide dismutase (SOD) and TBARS (Thiobarbituric Reactive Substances) assays were performed using assay kits (Cayman Chemical, Ann Arbor, MI; product numbers 706002 and 10009055 respectively) following kit instructions and a plate reader (SpectraMax) and using kit-provided standards for quantitation. Plasma samples were isolated from cattle venous blood samples after centrifugation and immediately frozen at -80 ⁰C before analysis, with only a single freeze-thaw cycle, and were diluted to the linear range of the assay for quantitation.

**References for Additional File 1**

[1]. Vrentas CE, Onstot S, Nicholson EM. A comparative analysis of rapid methods for purification and refolding of recombinant bovine prion protein. Protein Expn Purif. 2012;82*:*380-382.

[2]. Greenfield NJ. Using circular dichroism collected as a function of temperature to determine the thermodynamics of protein unfolding and binding interactions. Nat Protoc. 2006;1:2527-2535.

[3]. Pace CN, Shirley BA, Thomson JA. Measuring the conformational stability of a protein. In: Creighton T, editor. Protein Structure: A Practical Approach. Oxford: IRL Press; 1989. p. 311‐329.

[4]. Wildegger G, Liemann S, Glockshuber R. Extremely rapid folding of the C- terminal domain of the prion protein without kinetic intermediates. Nat Struct Biol. 1999;6:550-553.

[5]. Greenfield NJ. Determination of the folding of proteins as a function of denaturants, osmolytes or ligands using circular dichroism. Nat Protoc. 2006;1:2733-2741.

[6]. Brunelle BW, Greenlee JJ, Seabury CM, Brown CE 2^nd^, Nicholson EM. Frequencies of polymorphisms associated with BSE resistance differ significantly between *Bos taurus*, *Bos indicus*, and composite cattle. BMC Vet Res. 2008;4:36.

[7]. Carr IM, Robinson JI, Dimitriou R, Markham AF, Morgan AW, Bonthron DT. Inferring relative proportions of DNA variants from sequencing electropherograms. Bioinformatics. 2009;25:3244-3250.

[8]. Msalya G, Shimogiri T, Ohno S, Okamoto S, Kawabe K, Minezawa M, et al. Evaluation of *PRNP* expression based on genotypes and alleles of two indel loci in the medulla oblongata of Japanese Black and Japanese Brown cattle. PLoS One. 2011;6:e18787.

[9]. Canello T, Friedman-Levi Y, Mizrahi M, Binyamin O, Cohen E, Frid K, et al. Copper is toxic to PrP-ablated mice and exacerbates disease in a mouse model of E200K genetic prion disease. Neurobiol Dis. 2012;45:1010-1017.
