# Supplementary material for: Effects of a naturally occurring amino acid substitution in bovine PrP: a model for inherited prion disease in a natural host species
Source: BMC Res Notes. 2017 Dec 20;10:759. doi: 10.1186/s13104-017-3085-8 (PMC5738711; doi:10.1186/s13104-017-3085-8)
Supplement: Supplementary file 2 — Additional file 2. Additional Statistical Analysis. Additional description of statistical tests completed on the data, potential statistical limitations and their implications, and prospective statistical power analyses for assessment of effects of the 200K mutation. [file 13104_2017_3085_MOESM2_ESM.docx]

**Additional File 2: Statistical Analysis Table**

|  | **1** | **2** | **3** | **4** | **5** | **6** | **7** | **8** |
| --- | --- | --- | --- | --- | --- | --- | --- | --- |
| **Test for Comparison** | **EE_211_ Average** | **EE_211_ Confidence Interval (95%)** | **EK_211_ Average** | **EK_211_ Confidence Interval (95%)** | **Student’s t-test p-value** | **Welch’s t-test p-value** | **Power:**  **# Animals needed for 40%** | **Power: # Animals needed for 10%** |
| *PRNP* Expression Levels (ΔC_t_) | 10.7 | 10.6-10.8 | 11.3 | 11.0-11.6 | 0.047 | 0.11 | -- | -- |
| TBARS Readings from Blood | 0.18 | 0.15-0.21 | 0.19 | 0.17-0.21 | 0.59 | 0.60 | 6 | 88 |
| SOD Levels in Blood (U/ml) | 0.043 | 0.036-0.050 | 0.052 | 0.044-0.060 | 0.16 | 0.16 | <6 | 62 |

The table above provides additional statistical analysis of the three types of quantitative data collected on the EE_211_ and EK_211_ cattle groups, listed under “Test for Comparison”.

**Columns 1-4**: Presentation of average and 95% confidence interval (CI) values for each cattle group, as also presented in the Main Text (data from Table 1).

**Columns 5 and 6**: Student’s and Welch’s t-test comparisons of the values from Columns 1 and 3; tests were performed with GraphPad software (LaJolla, CA).

The Welch’s t-tests utilized above do not require that the two groups for comparison have equal variances, but both t-tests do make the underlying assumption that the data are distributed normally. However, we do not have a large enough population of the EK_211_ cattle to complete a test of normality, or for the populations to be large enough for the t-test to perform well in the absence of normality. Therefore, this is a limitation that is inherent to small sample sizes associated with rare study populations, such as EK_211_ cattle, and limits our interpretations of the t-tests. Note that C_t_ values for qPCR analysis above are inherently log-transformed, which can be argued to aid in normality.

**Columns 7 and 8**: We utilized the data from the EE_211_ animals (observed mean and standard deviation) to compute the number of animals needed per group for sufficient power to detect an increase of either 40% (Column 7) or 10% (Column 8) in the average values in the EK_211_ population. This is a prospective calculation of power, meaning that we cannot retroactively apply the calculation to the current study, but it provides a means of presenting the numbers of cattle that would be expected to be needed to detect differences in future studies.

--Power calculations were performed with the ClinCalc tool (Kane SP. Sample Size Calculator. ClinCalc: http://clincalc.com/stats/SampleSize.aspx. Updated July 1, 2017. Accessed November 26, 2017).

--Parameters were set as follows: α = 0.05, Power = 80%, 1:1 ratio of sample sizes between groups. Numbers presented in the table reflect the total number of animals (50% in EE_211_, 50% in EK_211_).

We only have displayed the calculation for the two oxidative stress tests, as the *PRNP* expression data indicates that this comparison has sufficient power to generate a statistically significant difference in the case of the student’s t-test.
